# Novel Diagnosis Technologies for a Lack of Oil Lubrication in Gearmotor Systems, Based on Motor Current Signature Analysis

**DOI:** 10.3390/s22239507

**Published:** 2022-12-05

**Authors:** Mohamed Habib Farhat, Len Gelman, Gerard Conaghan, Winston Kluis, Andrew Ball

**Affiliations:** 1 Department of Engineering and Technology, School of Computing and Engineering, The University of Huddersfield, Huddersfield HD1 3DH, UK; 2 Daifuku Airport Technologies, Sutton Road, Hull HU7 0DR, UK; 3 Babcock International Group, Schiphol Boulevard 363, 1118 BJ Schiphol, The Netherlands

**Keywords:** gearbox, diagnostics, motor current signature analysis, signal processing

## Abstract

Due to the wide use of gearmotor systems in industry, many diagnostic techniques have been developed/employed to prevent their failures. An insufficient lubrication of gearboxes of these machines could shorten their life and lead to catastrophic failures and losses, making it important to ensure a required lubrication level. For the first time in worldwide terms, this paper proposed to diagnose a lack of gearbox oil lubrication using motor current signature analysis (MCSA). This study proposed, investigated, and experimentally validated two new technologies to diagnose a lack of lubrication of gear motor systems based on MCSA. Two new diagnostic features were extracted from the current signals of a three-phase induction motor. The effectiveness of the proposed technologies was evaluated for different gear lubrication levels and was compared for three phases of motor current signals and for a case of averaging the proposed diagnostic features over three phases. The results confirmed a high effectiveness of the proposed technologies for diagnosing a lack of oil lubrication in gearmotor systems. Other contributions were as follows: (i) it was shown for the first time in worldwide terms, that the motor current nonlinearity level increases with the reduction of the sgearbox oil level; (ii) novel experimental validations of the proposed two diagnostic technologies via comprehensive experimental trials (iii) novel experimental comparisons of the diagnosis effectiveness of the proposed two diagnostic technologies.

## 1. Introduction

Gearboxes are a key mechanical component widely used in various industries, such as energy (wind turbines), automotive and aerospace, etc. Lubrication is a crucial factor in the efficiency and operating lifetime of rotating components and improper lubrication could cause their faults/failures. Therefore, it is important to have proper levels of lubricants in gearboxes to insure the higher productivity, uptime, and efficiency of machines.

In most typical applications, gearmotor systems are exposed to high mechanical loads and wide temperature ranges which make the decrease of their lubrication oil levels common, either due to transformation in the oil composition (i.e., contamination by liquid or solid impurities) or due to sudden oil leaks that can be caused by loose plugs or crankcase perforation. Thus, it is important to have an on-line technique which can monitor the oil levels inside gearmotor systems.

On-line sensors that are directly installed in systems and continuously monitor oil conditions may offer a solution to these kinds of problems. Different approaches have been performed for this purpose, including impedance spectroscopy [1], oil debris sensors [2] and viscosity measurement [3]. An overview of the sensors currently used for wind turbine gearbox oil monitoring is given in [4], where the sensors are tested under different environmental conditions.

Motor current signature analysis (MCSA), which is a relatively new approach in condition-based maintenance (CBM), has been proven to be a reliable condition monitoring technique and has been the focus of many researchers in past years. The main reason is that induction motors (IM) are used in many types of machinery as they are easy to manufacture, cost-effective, reliable, and efficient. In addition, collecting stator current data is much simpler than other diagnostic techniques because no sensor should be mounted directly on the machinery and the price of the required equipment is lower.

MCSA is becoming a commonly used approach since it can be used to detect different types of faults in various components such as air-gap irregularities [5,6], broken rotor bars [7,8], cracked rotor end-rings [9], stator faults [10,11,12], abnormal connections of the stator windings [13], shaft eccentricity [14], and bearing and gearbox failures [15,16]. Overall, MCSA techniques can be divided into two categories.

The first category is the Park’s Vector Approach (PVA), which is a traditional method of MCSA [16,17]. This approach uses three phases of current data and maps them into a two-dimensional orthogonal space. Ideally, the resultant 2D representation has a circular pattern centered at the origin of the coordinates in unfaulty conditions. In the presence of damage, however, the thickness and shape of the representation differ. The variation in the pattern of the curve indicates a faulty component. A method has been proposed to further improve the sensitivity of this approach to the presence of damages and make PVA more suitable to incipient fault detection [18].

The second category analyses the spectral components in the frequency domain representation of stator current signals. Ref. [19] is one of the early attempts to employ MCSA to highlight the effect of a broken bar in the current signal spectrum. Refs. [20,21,22] show the possibility of detection of a motor shaft bearing damage of an induction motor. Ref. [23] performs a spectral analysis of the squared of current signals for rotor broken bars and rotor eccentricity diagnosis using simulation and experimental data.

Ref. [24] proposes Extended Park’s Vector Approach (EPVA) for stator winding faults diagnosis, i.e., EPVA computes the modulus of Park’s vector and investigate its frequency components. Ref. [25] combines EPVA and Hilbert transform to enhance the performance of EPVA. First, the Hilbert transform of the current for three phases are computed and then spectral analysis of the modulus of Park’s vector created by the analytic signals are examined for detection of the occurrence of shorted turns. The Park’s vector product approach (PVPA) is proposed in Ref. [26] to evaluate frequency components of the product of the real and imaginary parts of the Park’s vector to broken bars and short-circuit. Ref. [18] introduces filtered Park’s and filtered extended Park’s vector approach to identify broken rotor bars in induction motors. Filtered PVA is different from traditional PVA as some filtering processes are performed on a current signal to reduce the effect of the dominant supply frequency and other irrelevant components to a defect. In addition to using the 2D Filtered PVA plot to display the presence of damage, its spectrum is also considered for spectral analysis and fault-related frequency components analysis.

There are no works in the literature which have taken advantage of motor current signals analysis to diagnose a lack of oil lubrication in gearboxes. Ref. [27] detects a lack of lubrication in a gearbox of a wind turbine using generator current signature analysis (GCSA). The current signals are recorded for various levels of oil in the gearbox. Statistical features in the frequency domain representation of the signals are computed. Then a statistical model using k-nearest neighbors (KNN) is developed to capture the relationship between the frequency domain features of the current signals and the amount of oil inside the gearbox. In addition, the accuracy of the correct classification is calculated for various combinations of the features to find the most informative ones. Ref. [28] employs MCSA to estimate the viscosity of oil in a gearbox. Afterwards, the variation of the estimated viscosity is used for condition monitoring and diagnosis of the gearbox, as the viscosity has a considerable effect on the mechanical and thermal characteristics of the machinery.

Despite all the promotive results for MCSA in machinery defect diagnosis, there are no technologies in the existing literature and on the market for diagnosing a lack of gear oil lubrication via MCSA.

This paper proposed and investigated, for the first time in worldwide terms, two novel powerful diagnostic technologies for oil level diagnosis in gearmotor systems based on MCSA. Two diagnostic features were proposed and applied for the first time in this study to diagnose oil level in a gearmotor, based on MCSA. The effectiveness of the proposed technologies was evaluated using the current signals, recorded for different oil levels in a three-phase induction gearmotor used to drive a conveyor belt system for a baggage handling at airports. To investigate the effect of grid fluctuations, two sets of data, corresponding to two different measurement days (Day 1 and Day 2), were considered as a reference for the standard oil level condition. The study separately used three phases of motor current signals and diagnostic features, which were then averaged over three phases.

The main difference between the approaches/technologies proposed in this paper, and the technology in Ref. [27] is that the technology in Ref. [27] uses GCSA, while this paper proposed to use MCSA for diagnosis of a gear lack of lubrication. Furthermore, in Ref. [27], the classical simple time domain-based diagnostic features, such as the standard deviation, the kurtosis, and the median, are used to diagnose a gear lack of lubrication. The physical interpretations of these features are very limited in Ref. [27]. Therefore, the authors underwent a comprehensive training phase for the classifier before they could diagnose a gear lack of lubrication, and the obtained results were strongly dependent on a training dataset, which limited the effectiveness of their method. In contrast, in this work, the proposed technologies have a clear physical interpretation and can be used to diagnose an oil level in any gearmotor with a very limited training phase/data.

The main novelty of the paper is a novel proposition (by L. Gelman) for the first time in worldwide terms, for performing the diagnosis, via MCSA, of a lack of oil lubrication of gearboxes coupled to induction motors.

Other novelties presented in this paper included:

- The proposed novel diagnostic technology/feature, which employs the power in a frequency range around the fundamental harmonic and the higher harmonics of the supply frequency;
- It was shown for the first time in worldwide terms, that the energy consumption of a motor changes with the reduction of the gearbox oil level;
- The proposed novel diagnostic technology/feature, which employed spectral magnitudes of the fundamental harmonic of the supply frequency, normalized by the average value of the spectral magnitudes of higher harmonics of the supply frequency in the spectrum of the current signal;
- It was shown for the first time in worldwide terms, that motor current non-linearity level increases with the reduction of gearbox oil levels;
- Novel experimental validations of the proposed two diagnostic technologies were presented via comprehensive experimental trials;
- Novel experimental comparisons of the diagnosis effectiveness of the proposed two diagnostic technologies were presented.

The objectives of this paper were to:

- develop and investigate for the first time in worldwide terms two motor current-based diagnosis technologies for a lack of oil lubrication diagnosis in gearboxes, connected to induction motors;
- perform an experimental validation of the proposed technologies in diagnosing a lack of oil lubrication in gearboxes via comprehensive experimental trials;
- perform a comparison between the proposed diagnostic technologies;
- develop a strategy for diagnosing the lack of oil lubrication in gearboxes based on the obtained results.

This paper is structured as follows. In Section 2, two diagnostic technologies are proposed for diagnosing a lack of oil lubrication in gearmotor systems based on MCSA. Section 3 presents the experimental setup as well as the experiments performed to validate the effectiveness of the proposed technologies for a lack of oil lubrication for gearboxes. Section 4 provides diagnostic results for the two proposed technologies and gives a comparison between their results. Finally, conclusions are summarized in Section 5.

## 2. Novel Diagnostic Technologies for a Lack of Gearbox Oil Lubrication

The objective of this section is to propose two novel diagnostic technologies for a lack of oil lubrication in gearmotor systems based on motor current signature analysis. The proposition of using the motor current signals for such diagnosis is based on the assumption that a lack of oil lubrication of a gearbox converts into changes of the electrical energy consumption of the motor coupled to it.

A lack of gearbox oil lubrication has an influence on motor mechanical load. Electrical motors, subjected to a load variation, tend to adapt their produced electrical torque to compensate for the variation of the mechanical load, which results in a variation of their electrical energy consumption. Therefore, a method of diagnosing a lack of gear oil lubrication based on the power spectral density of supply harmonics in the gearmotor current signals was proposed.

The main idea of the proposed technology 1 is that a lack of gear oil lubrication at an initial oil reduction stage creates less motor load, and therefore reduces motor electrical energy consumption: i.e., less motor power is r0equired to circulate a reduced oil level inside a gearbox. This idea is justifiable provided that there is still enough oil to lubricate gear teeth and, therefore, there is still a liquid friction between gear teeth. If a solid–solid friction between gear teeth occurs as a result of removing more oil (i.e., at an advanced oil reduction stage), a motor energy consumption starts increasing.

If other factors will be affecting motor energy consumption, then more diagnostic features should be employed that will effectively differentiate a lack of gear oil lubrication from other factors.

The proposed technology 1 is based on Feature 1, which is the power in a frequency bandwidth around the harmonics of the supply frequency, as follows:

(1) $$\mathrm{Feature}\;1=\overset{f=f_{k}+\frac{B}{2}}{\underset{f=f_{k}-\frac{B\;}{2}}{\sum }}S\left(f\right)$$

where $S\left(f\right)$ is estimate of the power spectral density of the current signal at a discrete frequency f, B is a frequency bandwidth, that is centered around frequency $f_{k}=k*f_{s}$, k is harmonic number of the supply frequency $f_{s}.$

The main idea of the proposed technology 2 is the same as for technology 1: i.e,. a lack of gear oil lubrication creates less motor load, and, therefore, reduces motor electrical energy consumption. However, taking into account possible intensity fluctuations of the fundamental supply harmonic, it is proposed for technology 2 to essentially reduce the influence of these fluctuations by normalizing the intensity of the fundamental supply harmonic by the intensities of the higher supply harmonics. The proposed normalized feature characterizes a motor current level of non-linearity. Therefore, diagnostic technology 2, based on Feature 2 and characterizing the motor current non-linearity level, is proposed for the first time worldwide to diagnose a lack of gear oil lubrication in gearmotor systems.

The proposed Feature 2 is the magnitude of the fundamental harmonic of the supply frequency, normalized by the average magnitude of the higher harmonics of the supply frequency in the spectrum of the current signal, as follows:

(2) $$\;\mathrm{Feature}\;2=\frac{\left|X_{\;1*fs}\right|}{\;\;\;\;\frac{1}{\left(K-1\right)}\;*\;\;\sqrt[{\;}]{\sum_{k=2}^{K}{\left|X{\;}_{k*f_{s}}\right|}^{2}}}\;\;,$$

where $f_{s}$ is the supply frequency, $X_{k*fs}$ is the short time chirp-Fourier transform [29,30] of the current signal x(t) at the k^th^ harmonic of the supply frequency $f_{s}$, k is harmonic number of the supply frequency $f_{s}$, K−1 is the total number of the considered higher harmonics of the supply frequency.

The following steps are needed for estimation of Feature 2:

- The instantaneous frequency (IF) of the supply grid is estimated from the current signals, based on the Hilbert transform phase demodulation approach, Ref. [31];
- The magnitudes of the fundamental harmonic and the higher harmonics of the supply frequency are estimated based on the short time Chirp Fourier transform, Refs. [29,30], of the current signal;
- The feature is estimated based on the magnitudes of the supply frequency harmonics.

The time-frequency technique, the short time chirp-Fourier transform [29], is used for feature estimation for the proposed technology instead of Fourier transform to encompass a variation in the time of the supply frequency.

Feature 2 can be physically interpreted as a factor of non-linearity of the motor current signal. An increase of the magnitudes of the higher harmonics of the supply frequency at a constant or decreased magnitude of the fundamental harmonic of the supply frequency (i.e., the increase of the non-linearity of the motor current) will result in a decrease in the value of the feature.

## 3. Setup for Experimental Technology Validation

**Figure 1:**
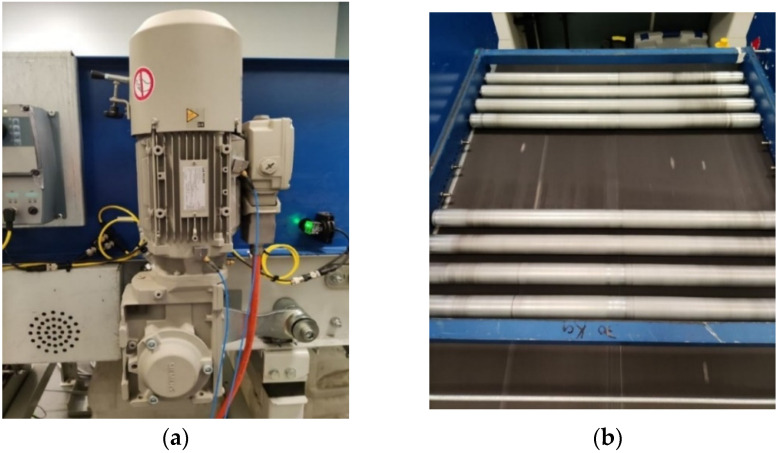
The experimental setup: (**a**) the gearmotor of the conveyor belt system; and (**b**) the loaded conveyor belt system.

The machinery under investigation is a conveyor belt system for a baggage handling at airports. A conveyor is driven by a gearmotor type Simens-JKE2104, consisting in a two stages gearbox, coupled to a three-phase AC induction motor (Figure 1a). The first stage of the gearbox consists of helical gears (30° helix angle) with a ratio of 18/37 (18-teeth pinion, 37-teeth gear) and the second stage consists of bevel gears with a ratio of 17/46 (17-teeth pinion, 46-teeth gear). The nominal power supply frequency of the motor is 50 Hz, and its nominal load and nominal speed are 40.6 Nm and 1440 rpm, respectively.

The experiments are carried out under no-load and under 20 kg load on the conveyor. The load consists of a series of rollers contained within a frame placed on the conveyor belt, as shown in Figure 1b.

**Figure 2:**
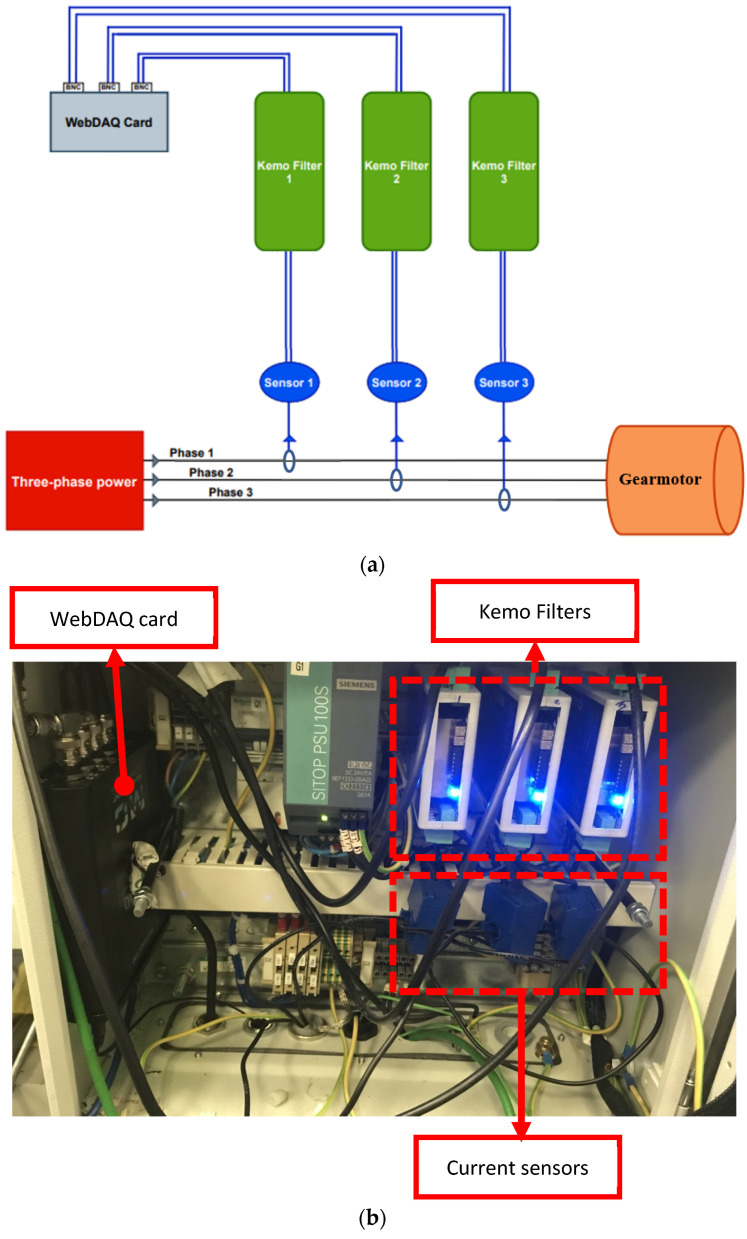
(**a**) schematic of the data acquisition system; and (**b**) the data acquisition system.

The data acquisition system and a schematic of that system are shown in Figure 2. The signals of three phases of the motor current are captured using LEM ATO-B10 current sensors. These sensors belong to the ATO series, with a frequency bandwidth of 1.5 kHz at level −1 dB. The sensors consist of a split-core current transformer used for the measurement of AC waveform currents, with galvanic separation between the primary circuit (power) and the secondary circuit (measurement). The rated primary current ($I_{\mathrm{Pr}}$) and voltage output of the sensors are 10 A and 333 mV, respectively.

The captured analogue current signals from each phase are passed through anti-aliasing filters of the KEMO DR 1600 type to avoid frequency aliasing during data sampling. The KEMO filter series is a small compact signal filter unit with switchable pre-filter input gain, wide range power input (9–30 Volts DC, 3 Wat), and a configurable input stage. The nominal attenuation rate of these KEMO filters in the transition band is 100 dB per octave, their bandwidth is 500 kHz, and their total harmonic distortion (THD) is lower than 0.003%. The KEMO filter also offers adjustable input gain in 1, 2, 5 steps to ×1000 (+60 dB), allowing the adjustment of the amplitude of the measured current signals to meet the input voltage range of the utilized data acquisition card. In the present study, the cut-off frequency, and the gain of the KEMO filters, are set to 8 kHz and ×20, respectively. The choice of 8 kHz cut-off frequency for the KEMO filters is because the frequency bandwidth of the selected current sensors is 1.5 kHz at level −1 dB, but 8 kHz at level −6 dB; therefore, 8 kHz cut-off frequency is selected in order not to miss an important current information. The gain is chosen considering the input voltage range of the acquisition card.

After limiting their bandwidth and choosing their appropriate gain, the motor current signals (output of the KEMO filters) are sampled at 51,200 Hz and recorded using a WebDAQ 504 data acquisition card. The sampling rate of 51,200 Hz is chosen since it is the maximum sampling rate for the WebDAQ 504 card. This maximum sampling rate ensures a more accurate estimation of the instantaneous frequency (IF) of the supply frequency, since at a higher sampling rate accuracy of frequency estimation is higher [31]. The WebDAQ 504 is a stand-alone vibration and acoustic logger designed for remote monitoring, analysis, and control. It offers 4 IEPE inputs, simultaneous sampling, 24-bit resolution and 4 digital I/O channels. The input frequency range of the WebDAQ 504 card is 13 MHz, its input voltage range is +/− 5 V.

The current signals of the motor are measured for different levels of oil in the gearbox. The first measurements are carried out under no-load conditions, where 10 signals of 20 min duration each are acquired using a brand new gearmotor containing 1600 mL of oil (i.e., the standard level of oil according to manufacturer’s specifications). Then, the load is set to 20 kg and signals are acquired for the standard oil level and when 120 mL, 260 mL, 280 mL and 490 mL of oil are removed from the gearbox. For each oil level (including the standard oil level), 10 signals of 20 min duration, acquired at the same acquisition date, are considered. For the case of the standard level of oil, 6 additional signals of 20 min duration, corresponding to a different acquisition date, are also considered. From now on, the standard oil level data acquired at two different measurement dates (under a 20 kg load) will be called Day 1 data and Day 2 data respectively.

## 4. Diagnosis Effectiveness of the Technologies and Technology Effectiveness Comparison

In this section, the proposed diagnostic technologies, based on Feature 1 and Feature 2, are experimentally validated for diagnosis of a lack of gearbox oil lubrication. Results will be presented separately for diagnostic technology, based on Feature 1 and diagnostic technology, based on Feature 2 and comparisons will be made between the results of the two proposed technologies.

### 4.1. Diagnosis Effectiveness of Technology 1

Feature 1 is estimated for a 1 Hz frequency band B, Equation (1), around the first and the third harmonics of the supply frequency. The selected frequency band is large enough to include the small variations of the supply frequency over time; however, it is not too large in order to isolate the supply harmonics from other characteristic spectral components in current spectrum.

For the multiple continuously acquired signals for 20 min and the different oil levels, every signal is divided into 50 s time segments with an overlap of 75% and then Feature 1 is estimated for each segment, while the power spectral density is estimated via a frequency resolution of 0.1 Hz. This process is repeated for three phases of current data. Frequency $f_{s}$ in Equation (1) is set to average of the IF of the supply frequency for each time segment, i.e., the IF is estimated using the Hilbert transform phase demodulation approach [31].

**Figure 3:**
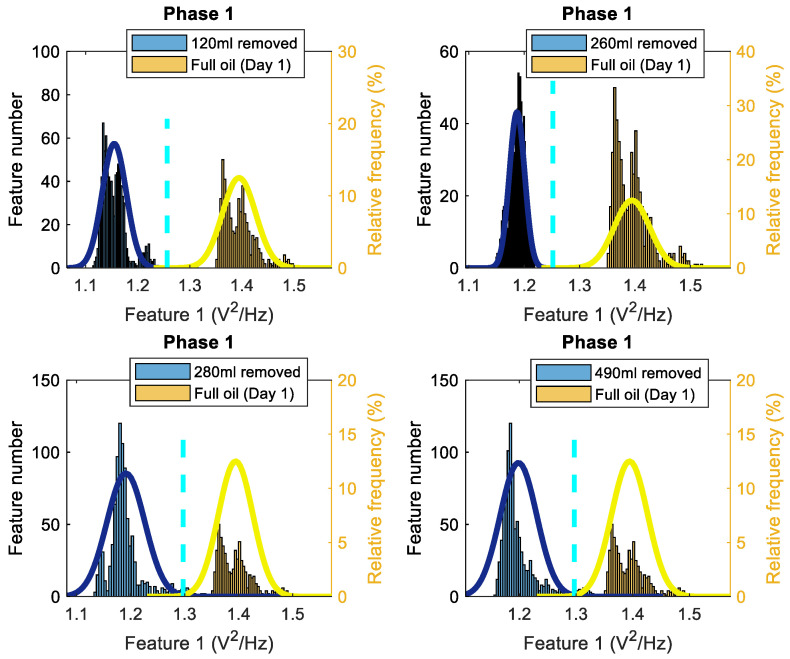
Histograms of Feature 1 (based on the first harmonic of the supply frequency) for phase 1 of the current signals, comparing the standard oil level (Day 1) to 120 mL, 260 mL, 280 mL and 490 mL of the removed oil cases (the solid lines are the PDF, and the dashed lines are thresholds).

After estimating Feature 1 for the standard oil level (Day 1), 120 mL, 260 mL, 280 mL, and 490 mL of removed oil cases, the estimates of the probability density functions of feature values for each case are evaluated via the histograms. Figure 3 displays four pairs of histograms, for the first phase of the current data, related to the removed oil cases and to the standard oil level (Day 1) case. It can be noticed that all levels of oil removal have lower feature values compared to the standard oil level and every two histograms are clearly separated. The same behavior could be seen while considering Feature 1 for phase 2 and phase 3, and when performing the same process as for Feature 1 for phase 1.

To consider the influence of an oil removal on three phases simultaneously, it is proposed here to employ average of Feature 1 for three phases. The results are similar to the other phases and there is a clear distinction between the 4 oil removal cases and the case in which the gearbox is working with the standard level of oil. The results show that a motor energy consumption reduces when oil is removed. The highest level of Feature 1 occurs when the gearbox is working with the standard oil level (Day 1) and for all other cases of oil removal, lower quantities are achieved for Feature 1. Hence, the energy level of the first harmonic of the supply frequency has the potential for monitoring/diagnosis of the oil level in gearboxes.

These histograms clearly highlight the main idea of technology 1, that a lack of gear oil lubrication at initial oil removal stage creates less motor load, and therefore reduces motor electrical energy consumption: i.e., less motor power is required to circulate reduced oil inside the gearbox, and therefore less average power is in a frequency range around the fundamental harmonic of the supply frequency (i.e., lower values of Feature1).

To estimate the total probability of correct diagnosis (TPOCD), which is the ratio of correctly diagnosed cases to the total number of cases for each two data sets presented in the histograms, firstly, a threshold should be defined. Afterwards, if the value of Feature 1 for an oil-removed case is lower than the defined threshold, then this oil-removed case is correctly diagnosed. Conversely, the standard oil case is correctly diagnosed once its Feature 1 value is higher than the defined threshold.

For one-dimensional Feature 1, the estimates of the probability density functions of this feature (Figure 3) are unimodal for the standard oil level case and for removed oil cases. Therefore, considering the unimodality of the estimates of the probability density functions of Feature 1, a simple and effective threshold-based decision-making rule is used via the Bayes criterion. Such a decision-making rule is effective and sufficient only for one-dimensional features with unimodal probability density functions and it is not the most accurate decision-making rule for other possible types of probabilities density functions even for one-dimensional diagnostic features (e.g. multimodal probabilities density functions). The advantage of the threshold-based rule is that the computational complexity is the lowest compared with other decision-making rules (e.g. neural network, etc.). This advantage is important for industrial applications. For more complicated probability density functions of Feature 1 and Feature 2, the implementation of a three-stage artificial intelligence decision-making rule [32,33] is proposed, including a k-nearest neighbor (KNN) anomaly detection method, a fault detection method and a fault diagnosis method.

To estimate a threshold for each pair of histograms of Feature 1, assuming, that Feature 1 has the normal distribution, the normal (i.e., the Gaussian) probability density functions (PDF) of the Feature 1 are estimated for every oil level. Next, an intersection point of two PDFs is utilized as the threshold value. The histograms, PDFs and the decision-making thresholds are depicted in Figure 3.

**Figure 4:**
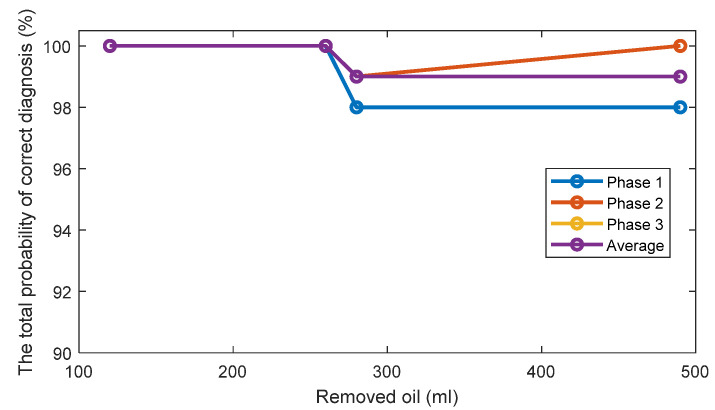
The TPOCD, provided by technology 1 (based on the first harmonic of the supply frequency) for three phases and 4 levels of oil removal compared to the standard oil level (Day 1).

The TPOCDs, using the above-mentioned decision-making procedure, are given in Figure 4 for all levels of the removed oil. The TPOCD shows a high effectiveness for three phases of the current data and for average feature.

The Fisher Criterion (FC) is defined as:

(3) $$\mathrm{FC}=\frac{{\left|m_{1}-m_{2}\right|}^{2}}{\sigma_{1}^{2}+\sigma_{2}^{2}}$$

where m and σ are the mean value and standard deviation of the feature values for each class of data, respectively, e.g., the standard oil level class and the removed oil classes.

The FC is an indicator of the level of separation between two classes of one-dimensional data. Thus, it is employed to quantify a separation between histograms of the standard oil level and of the removed oil cases.

**Figure 5:**
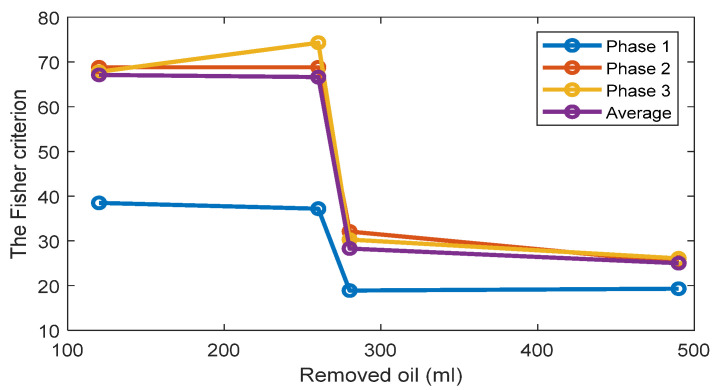
The FC of Feature 1 (the first harmonic of the supply frequency) for three phases and 4 levels of oil removal compared to the standard oil level (Day 1).

The FC are given in Figure 5 for all investigated cases. The results show that while the TPOCD are almost 100% for all cases, the features of phases 2 and 3 are better separated than the features of phase 1, as their FC are higher. Moreover, the level of separation between 120 mL or 260 mL cases and the standard oil level case (Day 1) is higher, compared to 280 mL or 490 mL of the removed oil cases. So, the effect of lower oil removal is more noticeable on the current signals, and while the amount of the removed oil increases, the effectiveness of the technology slightly decreases.

The TPOCD and the FC results reveal that at the early stage of oil removal, namely in the 120 mL and 260 mL cases, the separation between the standard oil level and the removed oil cases are higher, as a higher TPOCD and the FC are obtained. While the level of removed oil increases to 280 mL and 490 mL, the values of Feature 1 for these cases get closer to the standard oil level case, as a result, the TPOCD and the FC decreases. This is explained by the fact that, when a small amount of oil is removed at the beginning of the test, resisting force against the flow of the oil decreases; therefore, less motor power is required to circulate oil inside the gearbox resulting in reduction of Feature 1 values.

Further increase in the power usage of the electric motor by removing more oil, which leads to a lower TPOCD and the FC, is due to an improper lubrication of the gears. This is because by removing more oil, part of the liquid friction is transferred to a solid–solid friction between gear teeth; thus, more motor power is required due to solid–solid gear tooth friction.

**Figure A1:**
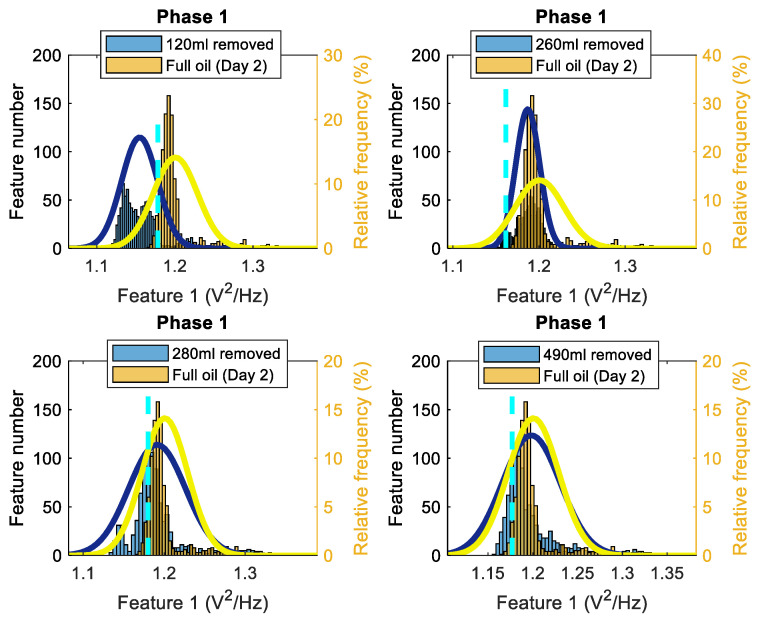
Histograms of Feature 1 (the first harmonic of the supply frequency) for phase 1 of the current signals, comparing the standard oil level (Day 2) to 120 mL, 260 mL, 280 mL and 490 mL of removed oil cases.

**Figure A2:**
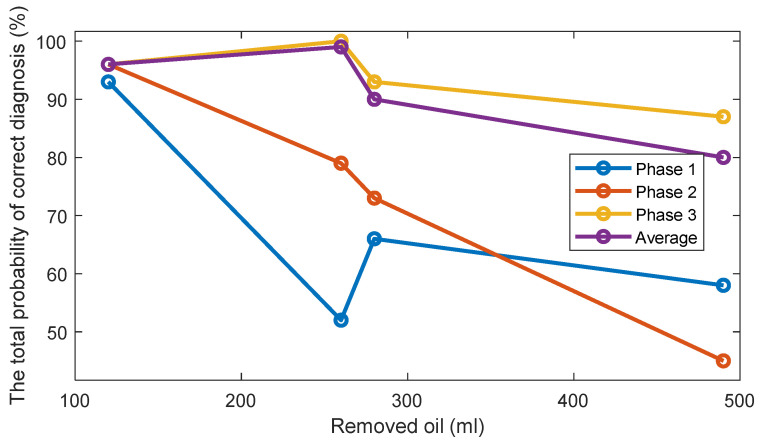
The TPOCD, provided by technology 1 (the first harmonic of the supply frequency) for 3 phases and 4 levels of oil removal compared to the standard oil level (Day 2).

To further investigate the effect of oil removal on the first harmonic of the supply frequency, standard oil level data is considered for a different day of the experiment (Day 2). Similar to the previous standard oil level data (Day 1), the values of Feature 1 for 120 mL, 260 mL, 280 mL and 490 mL are compared to the standard oil case (Day 2) for three phases. The histograms are depicted in the Appendix A, Figure A1 for phase 1, where here and in the whole paper, Figures with notation “A” are depicted in the Appendix A. In contrast to Day 1, Feature 1 for Day 2 data shows lower values and, therefore, its probability density function moves toward the “removed oil” probability density function, and, as a result, the overlap between two distributions increases. The TPOCD, considering Day 2 data, for the 4 levels of oil removal and three phases of current signals are given in Figure A2.

**Figure A3:**
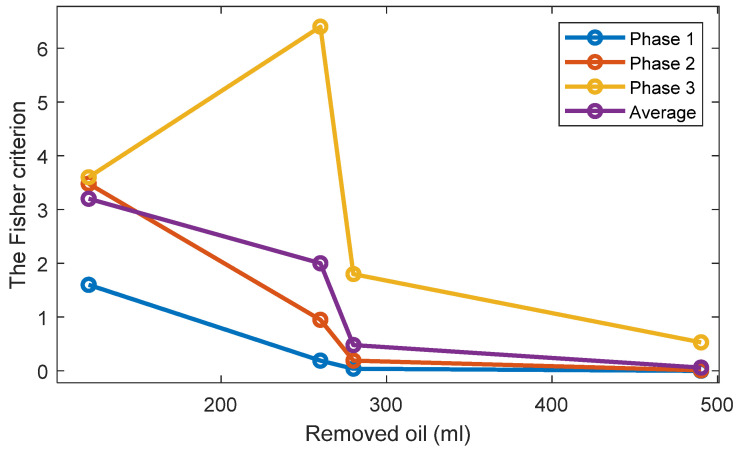
The FC of Feature 1 (the first harmonic of the supply frequency) for 3 phases and 4 levels of oil removal compared to the standard oil level (Day 2).

The TPOCD decreases in comparison to the ones. obtained for Day 1, though, in most cases, the two distributions are still separable. The highest TPOCD is achieved by using current signals of phase 3 among the three phases; the average of Feature 1 for all phases also provides a high TPOCD. The FC is given in Figure A3; because of the high level of overlap between distributions, low FC values are obtained in comparison with Day 1 data.

Overall, while the average of Feature 1 over three phases does not always offer the highest TPOCD for each condition, it is the most reliable indicator as it integrates all information of three phases, related to oil removal, and, therefore, high TPOCDs are achieved even when one of the phases does not provide enough information to properly distinguish between the standard oil level case and the removed oil cases. For instance, for Day 2 and 260 mL of the removed oil case, the TPOCD of 99% is obtained for the average feature, which is higher, than the 52% and 79% of the TPOCD, achieved for phases 1 and 2, respectively. Considering the average feature, the highest TPOCD is obtained for the 260 mL case and, after that, 120 mL, 280 mL, and 490 mL cases provide less level of the TPOCD.

**Figure A4:**
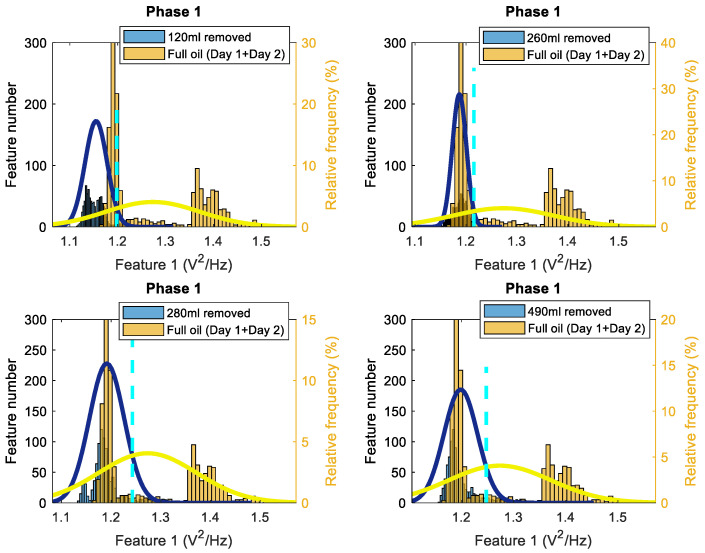
Histograms of Feature 1 (the first harmonic of the supply frequency) for phase 1 of the current signals, comparing the standard oil level (Day1 + Day 2) to 120 mL, 260 mL, 280 mL and 490 mL of removed oil cases.

**Figure A5:**
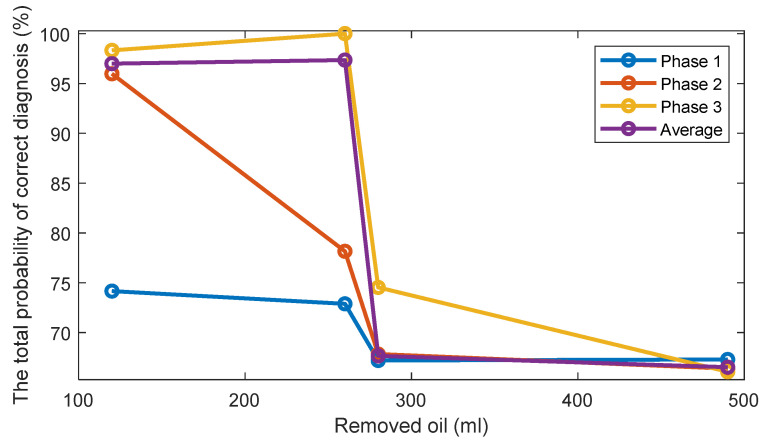
The TPOCD provided by technology 1 (the first harmonic of the supply frequency) for 3 phases and 4 levels of oil removal compared to the standard oil level (Day 1 + Day 2).

**Figure A6:**
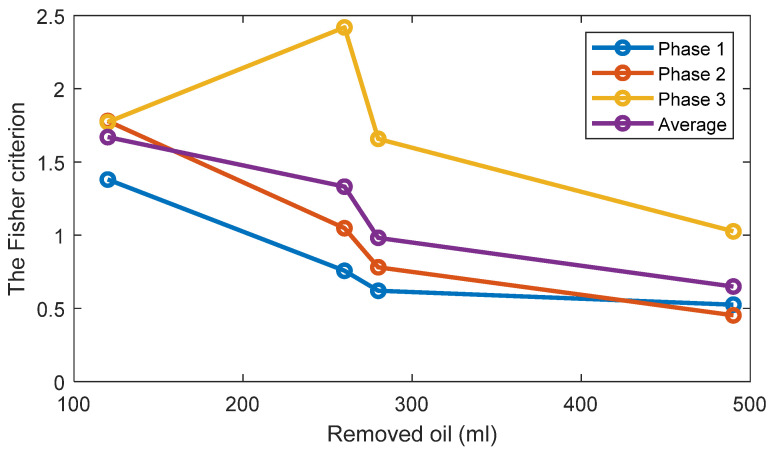
The FC of Feature 1 (the first harmonic of the supply frequency) for 3 phases and 4 levels of oil removal compared to the standard oil level (Day 1 + Day 2).

The results show that, while the energy level of the first harmonic of the supply frequency has some potential to diagnose different levels of oil inside the gearbox, it could also vary for reasons other than the oil level; as it is shown, Feature 1 values change while considering standard oil level data acquired in two different days. To show this variation, Feature 1 for the standard oil level data (Day 1 + Day 2) are compared to the removed oil cases. The histograms are depicted in Figure A4 for phase 1. There are two separated data clusters in the feature distribution for standard oil level data, which are associated with the different days of the standard oil level experiment. In addition, it can be seen that the assumption of the normal probability density functions for the distribution of Feature 1 is invalid under this circumstance. The TPOCD and the FC for three phases and 4 levels of oil removal compared to standard oil level data (Day 1+Day 2) are shown in Figure A5 and Figure A6, indicating that 120 mL and 260 mL of removed oil cases are more effectively diagnosed compared to 280 mL and 490 mL of removed oil cases and Feature 1 of phase 3 and average Feature 1 over three phases provide a higher TPOCD in comparison to Feature 1 of phases 1 and 2.

The first harmonic of the supply frequency is by far the strongest harmonic. The recorded current signals also include other harmonics of the supply frequency, but the magnitudes of the even harmonics are negligible. As was mentioned, Feature 1, estimated for the first harmonic, shows some variations when the data recorded for different days are used. In the following section, the TPOCD and the FC will be estimated for the third harmonic of the supply frequency to investigate diagnosis information it contains regarding the removed oil levels.

**Figure 6:**
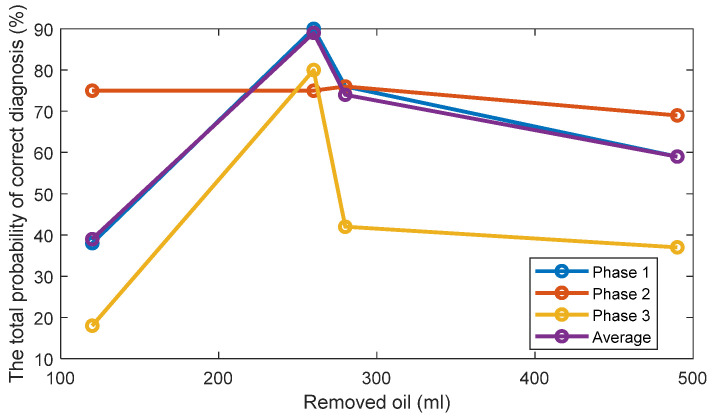
The TPOCD, provided by technology 1 (the third harmonic of the supply frequency), for three phases and 4 levels of oil removal compared to the standard oil level (Day 1).

**Figure 7:**
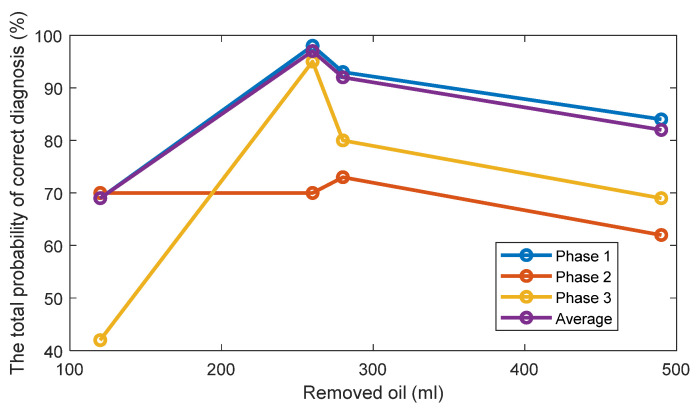
The TPOCD, provided by technology 1 (the third harmonic of the supply frequency) for three phases and 4 levels of oil removal compared to the standard oil level (Day 2).

**Figure 8:**
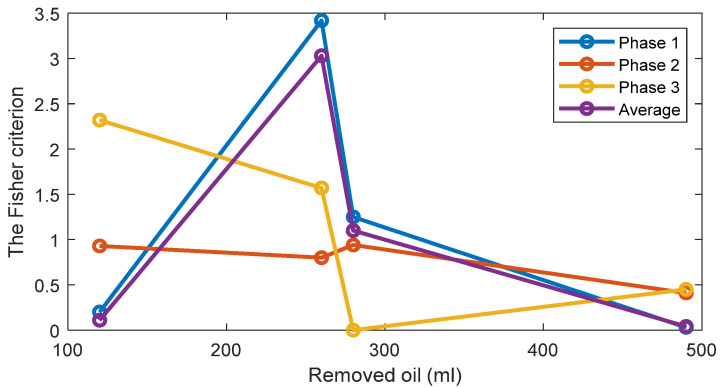
The FC of Feature 1 (the third harmonic of the supply frequency) for three phases and 4 levels of oil removal compared to the standard oil level (Day 1).

**Figure 9:**
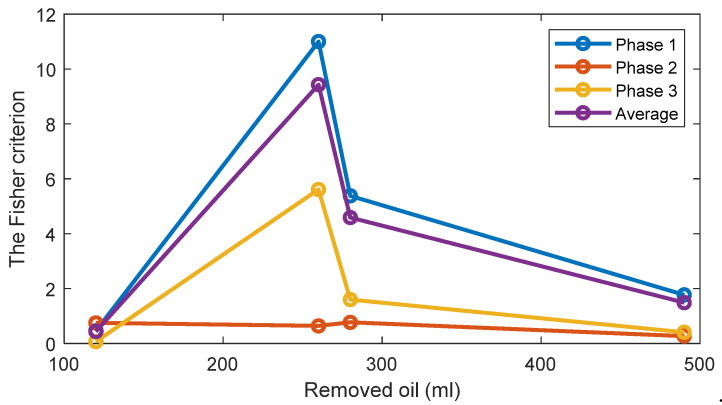
The FC of Feature 1 (the third harmonic of the supply frequency) for three phases and 4 levels of oil removal compared to the standard oil level (Day 2).

Considering Day 1 and Day 2 data, the TPOCDs for different levels of the removed oil and three phases for the third harmonic of the supply frequency are displayed in Figure 6 and Figure 7, while the FC are shown in Figure 8 and Figure 9. The results for Day 1 show that the power of the third harmonic does not include the same amount of information regarding removed gear oil levels, if compared to the power of the first harmonic and its TPOCD is considerably lower. For instance, the 120 mL case is mostly not detectable using Feature 1 for phases 1 and 3; Feature 1 for phase 2 provides a 75% of the TPOCD, which is lower, than the TPOCD of 100%, obtained for Feature 1 for the first harmonic. In addition, Feature 1 for phase 3 is able mainly to detect 260 mL case to some extent, and fails to diagnose most cases for the other oil levels. In contrast, considering Day 2 data, Feature 1 for the third harmonic provides comparable outcomes to Feature 1 for the first harmonic in most cases and even outperforms it in some cases, such as for phase 1 and 260 mL, 280 mL, and 490 mL of the removed oil. Although it should be mentioned that, using Feature 1 for the third harmonic, most of the data for Day and Day 2, related to 120 mL case, are not diagnosed correctly. Considering the average feature, the highest TPOCD is achieved for 260 mL case while 120 mL case has the lowest TPOCD for both Day 1 and Day 2. In addition, the TPOCD for 280 mL of the removed oil case is higher than for 490 mL of the removed oil case.

### 4.2. Diagnosis Effectiveness of Technology 2

To validate the diagnosis effectiveness of technology 2, effectiveness is estimated from the current signals, acquired for the different oil level cases. The acquired 20-min current signals are divided into 100 s non-overlapping segments and Feature 2 is estimated for each segment following the steps explained in Section 2. Instead of considering the CFT of the 100 s segments for feature estimation, each 100 s segment is subdivided into two non-overlapping 50 s sub-segments and average of CFTs of both sub-segments is obtained, i.e., the employed frequency resolution is 0.02 Hz. The purpose of the averaging is to reduce variance of a feature estimate. The total number of the higher harmonics of the supply frequency is selected as 19 as magnitudes of the higher harmonics of order 20 and more are very low; so, they can be neglected to speed up a feature estimation.

**Figure 10:**
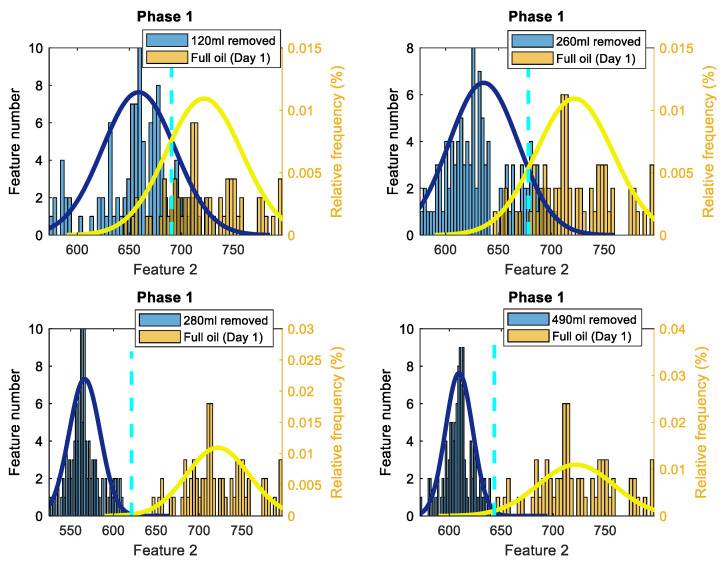
Histograms of Feature 2 for phase 1 of the current signals, comparing the standard oil level (Day 1) to 120 mL, 260 mL, 280 mL and 490 mL of the removed oil cases (the solid lines are the PDF, and the dashed lines are thresholds).

Following estimation of Feature 2 from the current signals corresponding respectively to the standard oil level (Day 1) and the 120 mL, 260 mL, 280 mL, and 490 mL of oil removal cases, estimates of the probability density functions of feature values are evaluated and compared, using histograms. Figure 10 shows histograms of Feature 2, extracted from the current signals of phase 1, for the different oil removal cases, with respect to the standard oil level case (Day 1).

From the histograms, it can be noticed that Feature 2 values are decreasing (i.e., motor current non-linearity is increasing) as oil is removed. However, at 120 mL level of removal, overlaps are observed between histograms for all phases. The overlaps become less frequent in the case of 260 mL of the removed oil for histograms of Feature 2 for phase 1 and phase 2 and no overlap is seen for the histograms of phase 3. For the cases of 280 mL and 490 mL of the removed oil, a complete separation is observed with respect to the standard oil level (Day 1) in all appropriate histograms. It means, that for 280 mL and 490 mL of the removed oil, the estimates of the TPOCD are 100% for all phases. Overall, Feature 2 for phase 3 shows better separation compared to Feature 2 for phase 1 and phase 2.

To make technology 2 more stable (robust) in conditions of possible variations of phase currents, it is proposed here to average values of Feature 2 over three phases.

These histograms clearly highlight the main idea of technology 2, that a lack of gear oil lubrication at initial oil removal stage creates less motor load, and, therefore, reduces motor electrical energy consumption: i.e., less motor power is required to circulate reduced oil inside the gearbox and, therefore, it is:

- decreasing magnitude of the fundamental supply frequency harmonic;
- increasing magnitudes of the higher supply frequency harmonics;
- increasing motor current level of non-linearity (i.e., less values of Feature 2).

**Figure 11:**
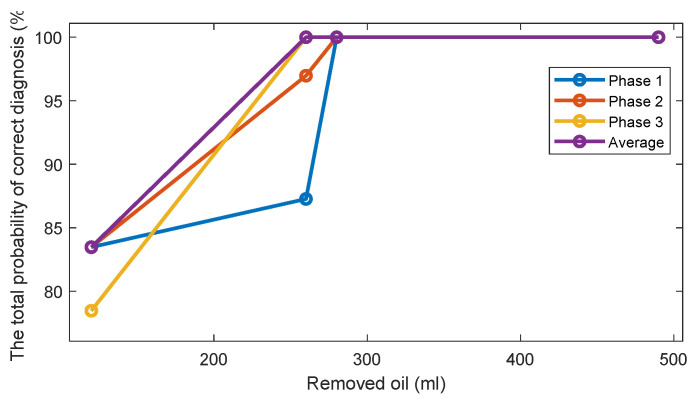
The estimates of the TPOCD, provided by technology 2 for three phases and 4 levels of oil removal compared to the standard oil level (Day 1).

**Figure 12:**
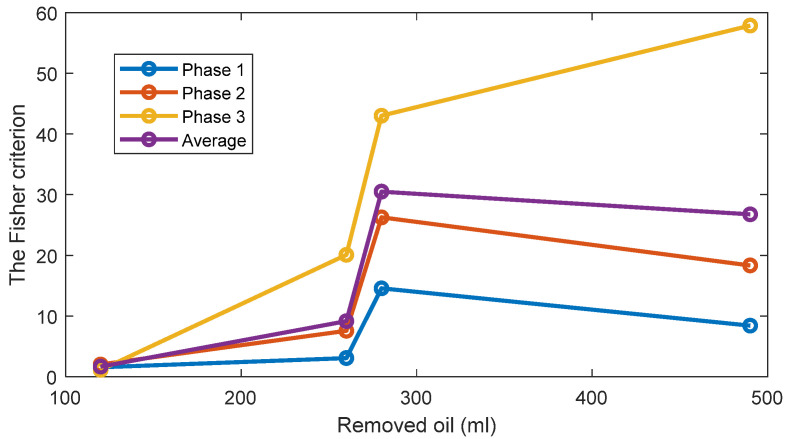
The FC of Feature 2 for three phases and 4 levels of oil removal compared to the standard oil level for Day 1.

For one-dimensional Feature 2, the estimates of the probability density functions of this feature (Figure 10) are also unimodal for the standard oil level case and for removed oil cases. Therefore, taking into account this unimodality property, a simple and effective threshold-based decision-making rule is also employed via the Bayes criterion. Assuming, that Feature 2 has the normal distribution, the normal (i.e., the Gaussian) probability density functions (PDF) of the Feature 1 are estimated for every oil level. The estimates of the TPOCD and the FC are evaluated, based on the histograms of Feature 2, and results are summarized in Figure 11 and Figure 12 respectively.

For the case of 260 mL of the removed oil, Feature 2 of phase 3 offers a better TPOCD than those of phase 1 and phase 2. In contrast, for the case of 120 mL of the removed oil, Feature 2 of phases 1 and 2 offers better TPOCD than those of phase 3 (i.e., higher TPOCD and the FC). Thus, Feature 2, estimated from phases 1 and 2, is more effective in the diagnosis of early stage of oil removal and Feature 2 estimated from phase 3, is becoming more effective as the amount of the removed oil increases. Averaging of Feature 2 across the three phases provides reasonable TPOCD for all levels of oil removal, but the TPOCD of phase 3 are still the best.

Altogether, feature averaging over the three phases could be a useful indicator since it provides good results even if Feature 2 for one of the phases (i.e., for phase 1 for 260 mL of the removed oil) provides not sufficient level of diagnosis effectiveness, which can be explained by a high correlation between features corresponding to the different phases.

**Figure A7:**
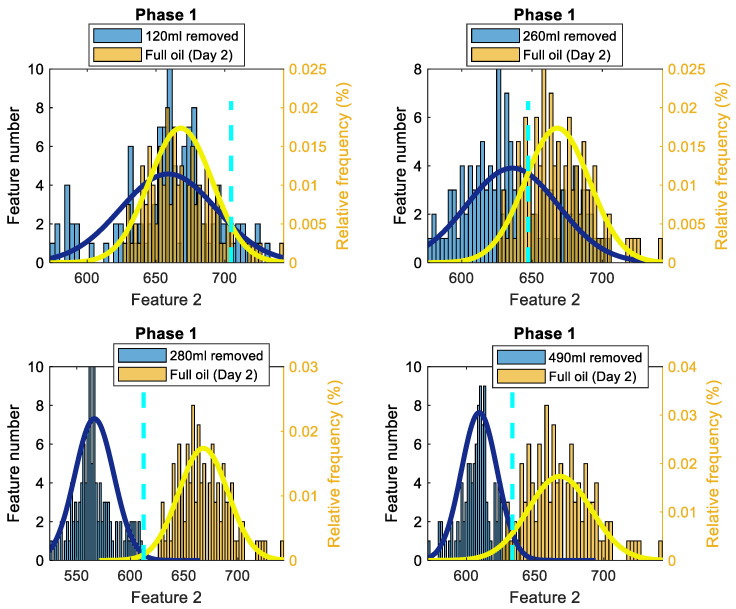
Histograms of Feature 2 for phase 1 of the current signals, comparing the standard oil level (Day 2) to 120 mL, 260 mL, 280 mL and 490 mL of removed oil cases.

To examine the effect of the current grid fluctuation on the performance of technology 2, the current signals, related to the standard oil level and collected on a different day of measurement (Day 2), are considered. Figure A7 shows histograms of Feature 2, extracted from the current signals of phase 1, for the different oil removal cases, with respect to the standard oil level (Day 2).

**Figure A8:**
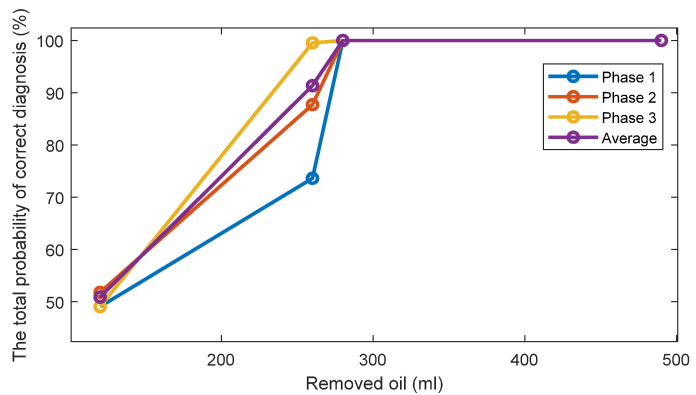
The TPOCD, provided by technology 2, for 3 phases and 4 levels of oil removal compared to the standard oil level (Day 2).

**Figure A9:**
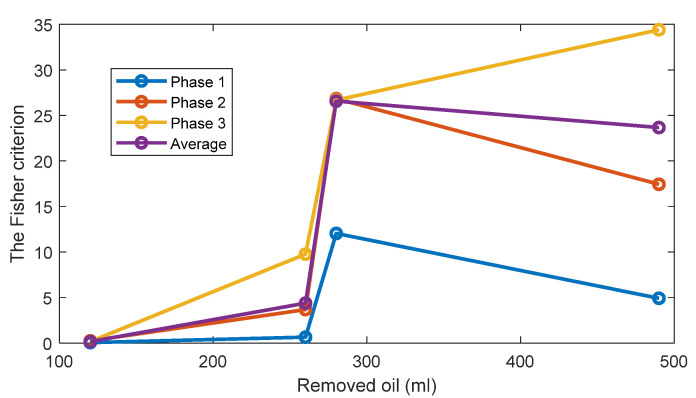
The FC of Feature 2 for 3 phases and 4 levels of oil removal compared to the standard oil level (Day 2).

It can be noticed from all histograms, that for Day 2, Feature 2 values are also decreasing (i.e., motor current non-linearity is increasing) as oil is removed. The TPOCD and the FC for the considered oil removals for Day 2 are presented in Figure A8 and Figure A9, respectively.

When using signals of Day 2 as a reference, an overlap is observed with data, corresponding to 120 mL oil removal, and a low TPOCD and the FC are obtained. Feature values tend to increase as 260 mL of oil is removed, but with an overlap for the data of Day 2. The feature overlap is greater for phase 1, and less for phase 3, making Feature 2 of phase 3 the most sensitive in the case of 260 mL of the removed oil. For the cases of 280 mL and 490 mL of the removed oil, a complete separation is observed with respect to data of Day 2 for all phases (meaning a 100% TPOCD), and Feature 2 of phase 3 offers the best separation between distributions (i.e., highest FC).

The results, obtained by averaging the three phases (averaged TPOCD and averaged FC), are better than those obtained when using Feature 2 for phase 1 and phase 2 separately, except for the case of 120 mL of the removed oil, where Feature 2 of phase 2 offers a slightly better TPOCD and the FC. On the contrary, Feature 2 for phase 3 shows better TPOCD and the FC than Feature 1, averaged over three phases for the 260 mL, 280 mL and 490 mL of the removed oil cases and lower performance in the case of 120 mL of the removed oil.

**Figure A10:**
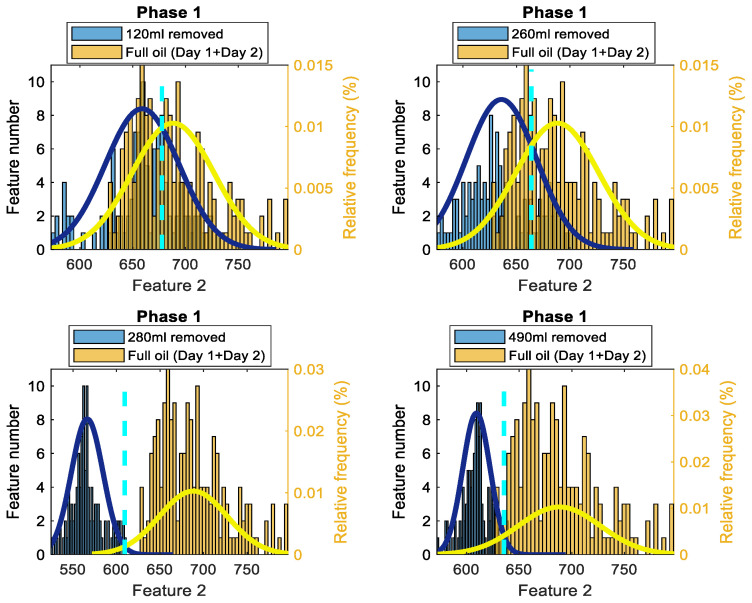
Histograms of Feature 2 for phase 1 of the current signals, comparing the standard oil level (Day 1 + Day 2) to 120 mL, 260 mL, 280 mL and 490 mL of removed oil cases.

**Figure A11:**
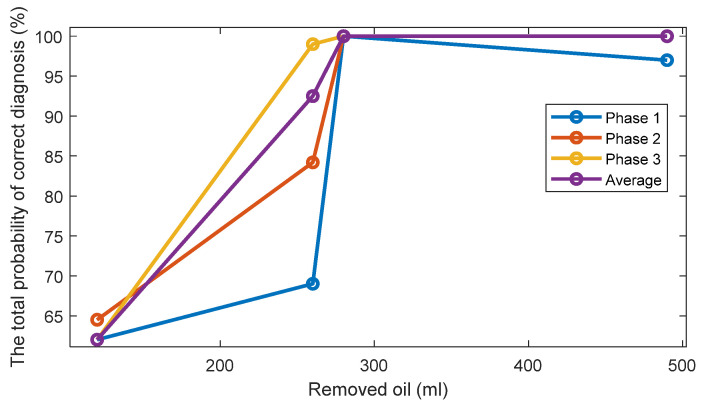
The TPOCD, provided by technology 2, for 3 phases and 4 levels of oil removal compared to the standard oil level (Day 1 + Day2).

**Figure A12:**
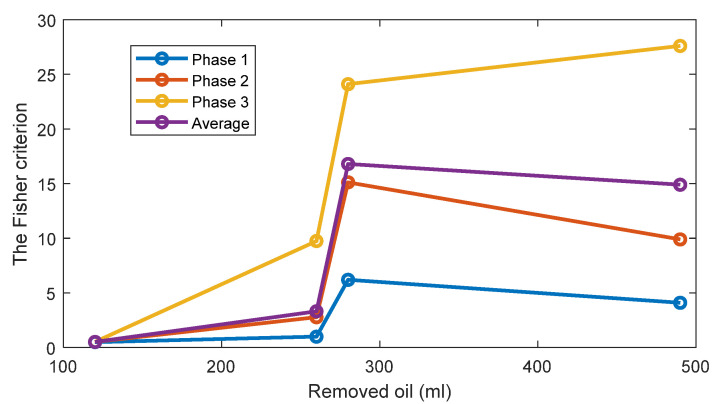
The FC of Feature 2 for 3 phases and 4 levels of oil removal compared to the standard oil level (Day 1 + Day2).

To evaluate the overall performance of technology 2 in diagnosis of a lack of gearbox oil lubrication, diagnosis is also performed using data of (Day1 + Day 2) as a reference for the standard oil level condition. Figure A10 shows histograms of the values of Feature 2, extracted from the current signals of phase 1, for the different oil removal cases, with respect to the standard oil level data of (Day 1 + Day 2). The TPOCD and the FC, related to the comparison of Feature 2 values for all the considered oil removals versus the standard oil level (Day 1 + Day 2), are summarized in Figure A11 and Figure A12, respectively.

When using signals of (Day 1 + Day 2) as a reference, a low TPOCD and the FC are obtained in the case of 120 mL of removed oil for three phases and when averaging over all phases. This is reflected for the histograms by a complete overlap between features for the standard oil condition (Day 1 + Day 2) and features for 120 mL of the removed oil. When 260 mL of oil is removed, Feature 2 of phase 3 offers the best TPOCD (98%), followed by Feature 2 of phase 2 (85%), then Feature 2 of phase 1 offers a poor diagnosis (68%), and the TPOCD of 92% is obtained for averaging Feature 2 across the three phases. Furthermore, a very good match is observed between the FC values and the degree of overlap (a visual interpretation) between the histograms, corresponding to the different phases, i.e., the lower the FC values, the higher the overlap, observed between histograms.

When 280 mL and 490 mL of oil is removed, Feature 2 for all phases and average of Feature 2 for all phases offer a high diagnosis effectiveness, i.e., all TPOCDs are 100%, except for phase 1, 490 mL; however, the TPOCD is also high, at 97%, for the last case.

Using Day 1 or Day 2 data separately, a 100% TPOCD is achieved for 280 mL and 490 mL of the removed oil from those of Day 1 and Day 2 signals for all phases. Feature 2 of phase 3 proved to be the most sensitive in the case 280 mL and 490 mL of the removed oil as it offers a better separation between the distributions compared to Feature 2 of phase 1 and phase 2.

While results of feature averaging across the three phases are better than results for Feature 2 for phase 1 and phase 2 for 260 mL, 280 mL and 490 mL the removed oil cases, results for Feature 2 for phase 3 are better, than results for average feature over three phases for most of oil removal cases, except for the case of 120 mL of the removed oil.

**Figure 13:**
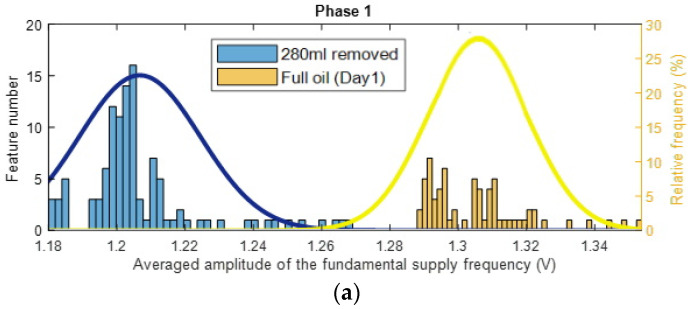

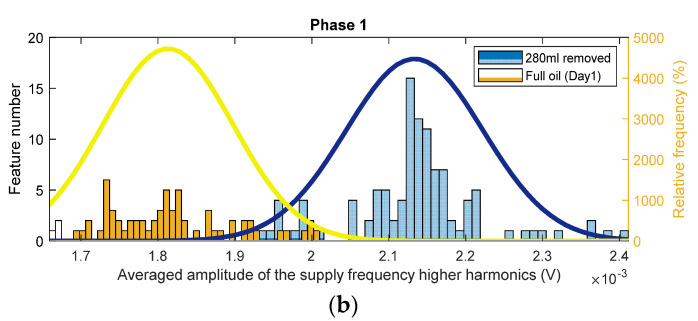
(**a**) Histograms of the average magnitude of the fundamental harmonic of the supply frequency; and (**b**) histograms of the average magnitude of the higher harmonics of the supply frequency. Data are corresponding to the standard oil level for Day1 and to 280 mL of the removed oil (20 kg load, phase 1).

As explained in Section 2, Feature 2 is the magnitude of the fundamental harmonic of the supply frequency normalized by the average magnitude of the higher harmonics of the supply frequency. To investigate the main reasons for the decrease of Feature 2 with oil removal (i.e., is this decrease due to the increase of the magnitudes of the supply frequency higher harmonics or/and due to the increase of the magnitude of the fundamental harmonic of the supply frequency), the average magnitude of the fundamental harmonic of the supply frequency and the average magnitude of the supply frequency higher harmonics are compared for the data, corresponding to the standard oil level (Day 1), and for the data corresponding to the case of 280 mL of the removed oil. Results are plotted in Figure 13.

From Figure 13, it could be noticed that, when 280 mL of oil are removed from the gearbox, the magnitude of the fundamental harmonic of the supply frequency is decreased (Figure 13a) and that the averaged magnitude of the higher harmonics of the supply frequency is increased (Figure 13b). Thus, it could be concluded that the decrease in Feature 2 with oil removal is the result of decrease of the magnitude of the fundamental harmonic of supply frequency as well as of increase of the magnitude of the higher harmonics of the supply frequency.

### 4.3. Diagnosis Efectiveness Comparision between Technologies 1 and 2

Based on the performed experimental tests, technologies 1 and 2 have proven to be effective in diagnosing a lack of oil lubrication in gearboxes, but with a varying performance depending on the amounts of the removed oil. To obtain a clear understanding of which technology offers better diagnostic performance on which amount of the removed oil, a comparison between their TPOCDs and their FCs in diagnosis of all the considered oil removals is performed for signals from the three phases.

**Figure 14:**
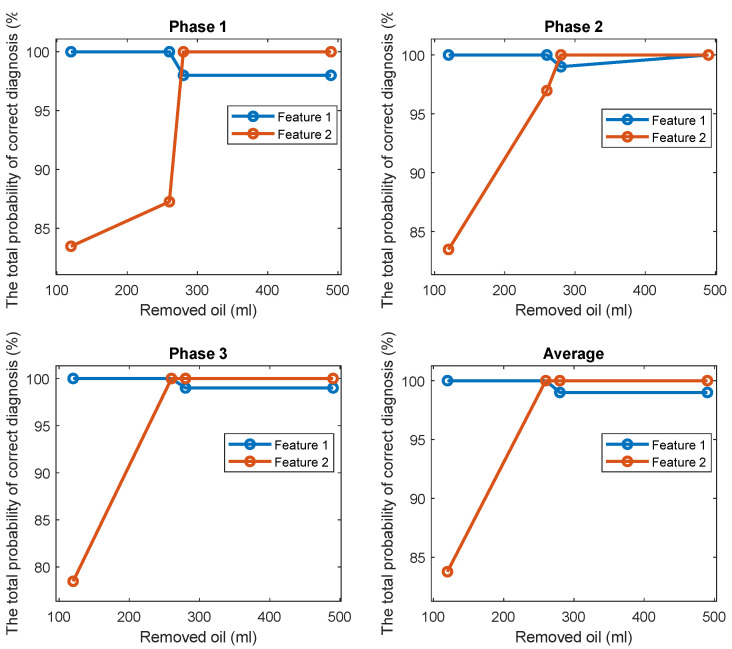
The TPOCD, provided by technology 1 and technology 2, respectively, for three phases and 4 levels of oil removal compared to the standard oil level for Day 1.

**Figure 15:**
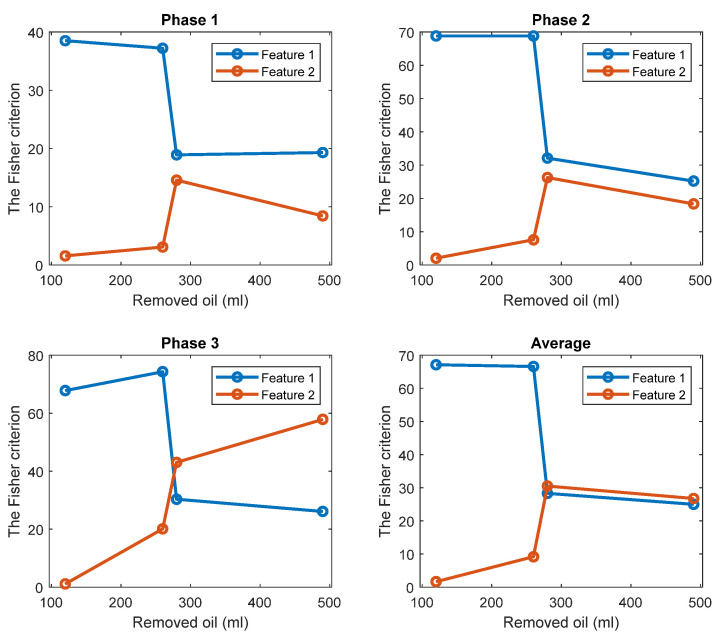
The FC of Feature 1 and Feature 2 respectively, for three phases and 4 levels of oil removal compared to the standard oil level (Day 1).

Figure 14 and Figure 15 display respectively a comparison between the TPOCD and the FC of technology 1 and technology 2 for data of Day 1 for the standard oil level condition. The results show, that, for the case of 120 mL of the removed oil, technology 1 presents a higher TPOCD and the FC, than technology 2, for the three phases and when averaging their features across all phases, making technology 1 the most sensitive, when removing small amounts of oil. The same behavior is noticed for the case of 260 mL of the removed oil, though with comparable TPOCD for technologies 1 and 2 in the case of phase 3 and the phase-averaging feature case.

When the amount of the removed oil reached 280 mL, the TPOCD of technology 1 still exhibits a high effectiveness. Technology 2 provides a 100% TPOCD for all cases. Technology 2 provides better TPOCD, compared to technology 1, in the case of 490 mL of the removed oil; Overall, technology 2 provides better diagnosis results, than technology 1, for the case of 490 mL of the removed oil, making technology 2 the most sensitive in the case of essential oil removal.

**Figure 16:**
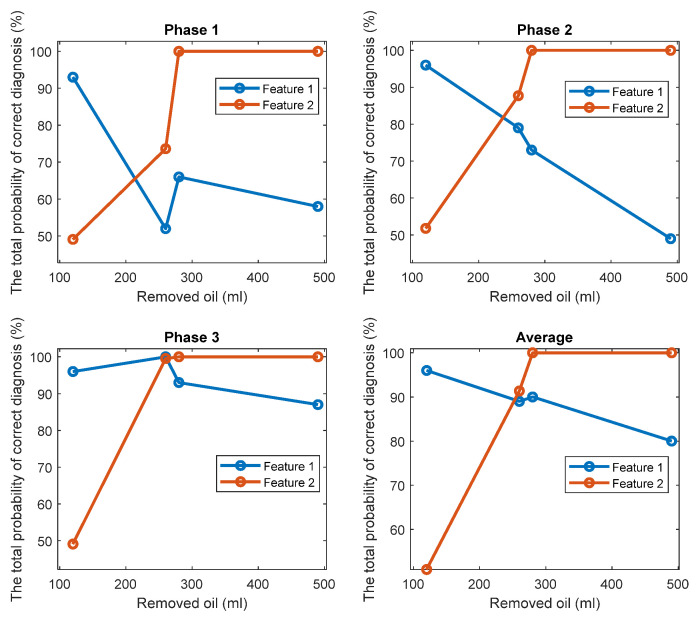
The TPOCD, provided by technology 1 and technology 2, respectively, for three phases and 4 levels of oil removal compared to the standard oil level (Day 2).

**Figure 17:**
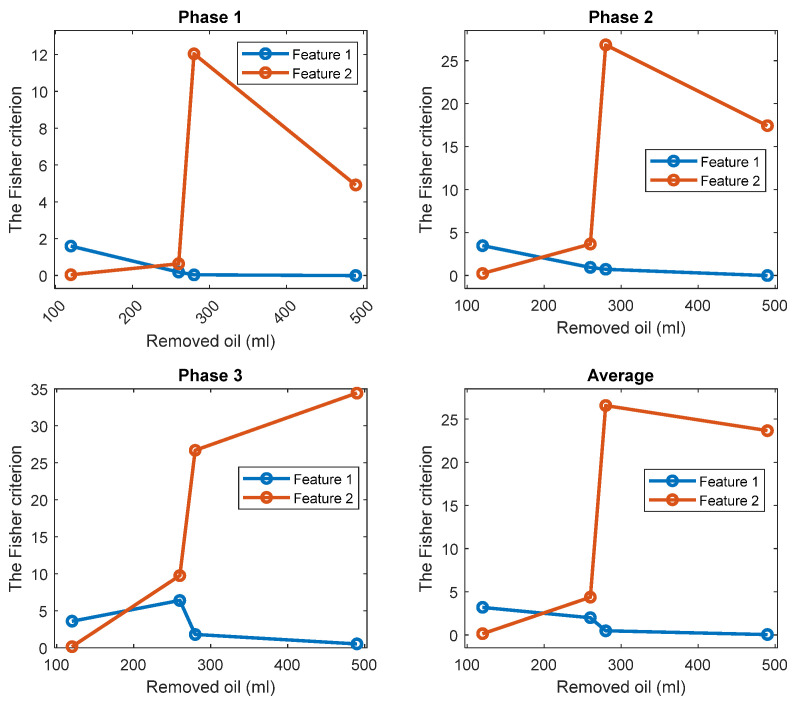
The FC for Feature 1 and Feature 2, respectively, for three phases and 4 levels of oil removal compared to the standard oil level (Day 2).

A second comparison between technology 1 and technology 2 is performed using data of Day 2 as a reference for the standard oil level condition. Figure 16 and Figure 17 show a comparison between the TPOCD and the FC of technologies 1 and 2 with respect to data of Day 2. Technology 1 provides the TPOCD and the FC higher than technology 2 for 120 mL of oil removal for three phases and when averaging features over all phases, confirming a higher sensitivity of technology 1 for diagnosing a small amount of oil removal. In the case of 260 mL of the removed oil, the FC of technology 2 are higher than those of technology 1, but none of the features offered a 100% TPOCD, except in the case of technology 1 for signals from phase 3.

Although the TPOCD of technology 2 is better than of technology 1 when considering signals from phase 1 and phase 2, technology 1 provides a better TPOCD, than technology 2, if considering phase 3 and if averaging features across all phases, making diagnosis results of technology 1 and technology 2 comparable in the case of a 260 mL of the removed oil. In cases of 280 mL and 490 mL of the removed oil, technology 2 provides higher TPOCD and higher FC than technology 1 for all cases. While the TPOCD of technology 2 is almost 100%, technology 1 presents a low TPOCD, especially, in the case of 490 mL of the removed oil, making technology 2 the most sensitive in the case of an essential oil removal.

Altogether, technology 1 provided better diagnosis results for the case of 120 mL of the removed oil and technology 2 provided better diagnosis results for 280 mL and 490 mL of the removed oil, with comparable effective diagnosis results for both technologies for 260 mL of the removed oil.

Based on these results, technology 1 is suggested for diagnosis of a reduction in gearbox oil level in relative oil reduction range (8–16%) and technology 2 is suggested for diagnosis of reduction in gearbox oil level in relative oil reduction range (16–31%). Relative oil reduction range (20–30%) is detrimental to the gearbox and requires urgent maintenance intervention.

It should be also noticed that technology 2 is less affected by the current grid fluctuations for three phases of current signals. In fact, while the overall decrease of Feature 1 for the Day 2 data compared to Day 1 data reached 15.2%, the overall decrease of Feature 2 for Day 2 data compared to Day 1 data did not exceed 6%. This could also be deduced from the diagnostic results (the TPOCD and the FC) which are, referring to Section 4.1 and Section 4.2, more stable between Day 1 and Day 2 for technology 2 compared to technology 1.

The difference between dependencies of technologies 1 and 2 on grid fluctuations could be explained by the fact, that Feature 2 is normalized while Feature 1 is not normalized. Indeed, the fluctuations of the current grid system are likely to influence the fundamental harmonic as well as the higher harmonics of the supply frequency. Thus, the normalization of the fundamental harmonic of the supply frequency by the higher harmonics of the supply frequency, adopted for Feature 2, provides less dependency of Feature 2 on current grid fluctuations, which is not a case for Feature 1, since it is unnormalized and it only considers the power of the harmonics of the supply frequency. Thus, for an application, in which current grid is fluctuating (which is usually the case for industrial applications), it is recommended to use technology 2 as a diagnostic technique for a lack of oil lubrication, rather than technology 1, to minimize the effect of current grid fluctuations on diagnostic effectiveness.

**Figure 18:**
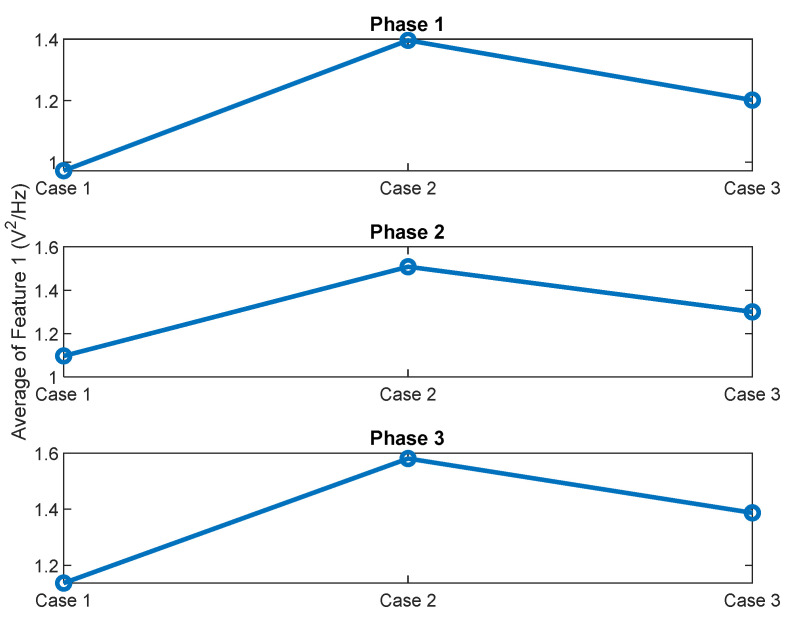
Average of Feature 1 for the three phases for the standard oil level for current signals, captured under different load conditions; Case1: no-load, Case2: 20 kg load (Day1) and Case 3: 20 kg load (Day2).

**Figure 19:**
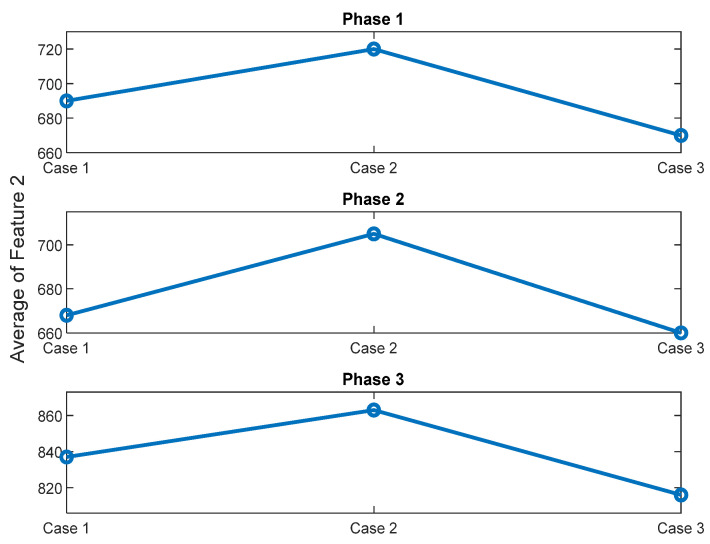
Average of Feature 2 for the three phases of the standard oil level for current signals, captured under different load conditions; Case1: no-load, Case2: 20 kg load (Day1) and Case 3: 20 kg load (Day2).

Another important advantage of the normalization, adopted for Feature 2, compared to Feature 1, is related to the case of a change in operating load of a conveyor for baggage handling, that is driven by a gearmotor. Figure 18 and Figure 19 show the average value of Feature 1 and Feature 2, respectively, for signals related to the standard oil condition and acquired under 20 kg load and no-load conditions for three phases of current signals.

It can be noticed from Figure 18 and Figure 19, that technology 1 is more affected, than technology 2, by change in a motor load. It can be seen from Figure 19, that for the three phases, value of average Feature 2, related to the no-load signals, is almost the same (i.e., 2–4.5% difference) as value of average Feature 2, related to 20 kg load signals for Day 1 and Day 2. Therefore, a decrease in operating load of a conveyor from 20 kg to no load will not essentially change Feature 2 and, therefore, will not trigger a false alarm of a lack of gearbox oil lubrication. This absence of false alarms is due to the normalization of this feature by the higher harmonics of the supply frequency.

In contrast, it can be seen from Figure 18 that the average Feature 1 for the no-load signals has lower quantit, than for 20 kg load signals for Day 1 and Day 2. Therefore, a decrease in an operating load of a conveyor from 20 kg to no load will lead to a decrease of electrical energy, consumed by the motor. This decrease will decrease Feature 1 and, therefore, may trigger a false alarm of a lack of gearbox oil lubrication.

Therefore, technology 1 could not be effectively used for a gear lack of oil lubrication diagnosis in conditions of essential current fluctuations in a grid system and of an essential variation of a conveyor operating load. For instance, Feature 1 for Day 1 and Day 2 of the experiment, which have the same standard level of oil, has different distributions, i.e., Feature 1 of Day 1 has higher values than Feature 1 of Day 2, and based on the approach, proposed in this paper, Day 2 should have a lower level of oil in gearbox compared to Day 1, which is not the case.

## 5. Conclusions

1. For the first time in worldwide terms, a method for performing the diagnosis of a lack of oil lubrication in gearboxes coupled to induction motors, via motor current signature analysis (MCSA) was proposed;
2. Two new diagnostic technologies for a lack of oil lubrication in gearboxes, based on MCSA, were proposed, investigated and experimentally validated;
3. Comprehensive experimental trials were performed for validation of the proposed two technologies. Three-phase motor current data of an AC induction motor driving a conveyor belt system (for baggage handling at airports) via a gearbox were recorded, respectively, for the standard oil level in the gearbox and for conditions in which specific amounts of oil are removed from the gearbox;
4. It was proposed that the gearbox oil level has an influence on the energy consumed by a motor. Thus, the power in a frequency bandwidth around harmonics of the supply frequency was proposed as diagnostic feature (Feature 1) for technology 1.
5. The experimental estimates of the probability density functions of Feature 1 values for the standard oil level and for the removed oil cases are evaluated and compared and the total probabilities of correct diagnosis (TPOCD) and the Fisher criteria (FC), based on these estimates, were also evaluated and compared. The study was carried out separately for the three phases of motor current signals and for the case of averaging Feature 1 over the three phases. The results showed that:
  - the power level of the first harmonic of the supply frequency is the most beneficial diagnostic feature as a higher TPOCD and the FC are obtained for this harmonic compared to the third harmonic of the supply frequency;
  - the power of the supply frequency harmonics changes as type of gear friction varies in the gearbox. When lower levels of oils are removed, there is still enough oil to lubricate gears via a liquid friction between gear teeth; as a result, a lower power is required to circulate oil inside a gearbox and, therefore, Feature 1 value decreases. When the solid–solid friction between gear teeth occurs as a result of removing more oil, a motor energy consumption starts increasing and Feature 1 value starts increasing as well;
  - technology 1 was shown to be sensitive/effective to the following oil removals: 120 mL (i.e., relative oil reduction is 8%) and 260 mL (i.e., relative oil reduction is 16%);
  - while using datasets corresponding to different days of experimental trials and different conveyor loads with a standard oil level, Feature 1 was shown to be affected by current fluctuations of the grid system and conveyor load variations.
  - it was shown that technology 1 could provide essentially different TPOCDs of a lack of gearbox oil lubrication for different current phases; therefore, it is recommended to employ three phases for diagnosis by technology 1;
  - Feature 1, averaged over the three phases, was proposed as a useful diagnostic feature, since it provides effective results even if one of the phases does not provide a sufficient level of diagnosis effectiveness.
6. It was proposed, for the first time in worldwide terms, that te non-linearity of the motor current changes due to a reduction of oil levels in gearboxes. Therefore, technology 2 was proposed, based on diagnostic Feature 2, for the diagnosis of a lack of gearbox oil lubrication. Feature 2 employs spectral magnitude of the fundamental harmonic of the supply frequency, normalized by the average value of the spectral magnitudes of higher harmonics of the supply frequency in the spectrum of the current signal and characterizes non-linearity of the motor current.
7. The experimental estimates of the probability density functions of Feature 2 values for the standard oil level and for the removed oil cases were evaluated and compared and the TPOCDs and the FCs, based on these estimates, were also evaluated and compared. The study was carried out separately for the three phases of motor current signals and for the case of averaging Feature 2 over the three phases. The results showed that:
  - non-linearity of the motor current increases with increase of an amount of the removed oil;
  - technology 2 was shown to be sensitive/effective to the following oil removals: 260 mL (i.e., relative oil reduction is 16%), 280 mL (i.e., relative oil reduction is 18%) and 490 mL (i.e., relative oil reduction is 31%).
  - while using datasets corresponding to different days of experimental trials and to different conveyor loads with standard oil levels, Feature 2 was shown to be almost unaffected by current fluctuations of the grid system and conveyor load variations.
  - it was shown that technology 2 could provide essentially different TPOCDs of a lack of gearbox oil lubrication for different current phases; therefore, it is recommended to employ three phases for diagnosis by technology 2.
  - Feature 2, averaged over the three phases, was proposed as a useful diagnostic feature, since it provided effective results even if one of the phases does not provide a sufficient level of diagnosis effectiveness.
  - It was shown that the increase in non-linearity of the motor current signals due to increase of oil removal results from both increase the magnitudes of the higher harmonics of the supply frequency and decrease of the magnitude of the fundamental harmonic of the supply frequency due to increase of oil removal.
8. Novel comparisons are made between the two proposed technologies. The following statements were concluded:
  - diagnostic Feature 1 of technology 1 is more affected by current fluctuations in a grid system and by variation of gearbox operating load compared to Feature 2 of technology 2, because Feature 1 is unnormalized. Therefore, technology 1 is recommended for use in conditions of no- to low-current fluctuations of a grid system and of no- to low-variations of a gearbox load to ensure a reliable diagnosis and to avoid false alarms;
  - diagnostic Feature 2 is less affected by current fluctuations in a grid system and by variation of gearbox operating load compared to Feature 1, because Feature 2 is normalized. Therefore, technology 2 is recommended to use in conditions of medium to high current fluctuations of a grid system and of medium to high variations of a gearbox load to ensure a reliable diagnosis and to avoid false alarms;
  - technology 1 has shown its ability for diagnosing a lack of gear oil lubrication with more sensitivity to training data, compared to technology 2.
